# Advances Towards How Meiotic Recombination Is Initiated: A Comparative View and Perspectives for Plant Meiosis Research

**DOI:** 10.3390/ijms20194718

**Published:** 2019-09-23

**Authors:** Ju-Li Jing, Ting Zhang, Ya-Zhong Wang, Yan He

**Affiliations:** National Maize Improvement Center, China Agricultural University, Beijing 100094, China; jjlcurie@cau.edu.cn (J.-L.J.); zhangting@cau.edu.cn (T.Z.); wangyazhong917@gmail.com (Y.-Z.W.)

**Keywords:** meiosis, double-strand break (DSB), hotspot, homologous recombination, DSB regulation, plants, chromatin

## Abstract

Meiosis is an essential cell-division process for ensuring genetic diversity across generations. Meiotic recombination ensures the accuracy of genetic interchange between homolous chromosomes and segregation of parental alleles. Programmed DNA double-strand breaks (DSBs), catalyzed by the evolutionarily conserved topoisomerase VIA (a subunit of the archaeal type II DNA topoisomerase)-like enzyme Spo11 and several other factors, is a distinctive feature of meiotic recombination initiation. The meiotic DSB formation and its regulatory mechanisms are similar among species, but certain aspects are distinct. In this review, we introduced the cumulative knowledge of the plant proteins crucial for meiotic DSB formation and technical advances in DSB detection. We also summarized the genome-wide DSB hotspot profiles for different model organisms. Moreover, we highlighted the classical views and recent advances in our knowledge of the regulatory mechanisms that ensure the fidelity of DSB formation, such as multifaceted kinase-mediated phosphorylation and the consequent high-dimensional changes in chromosome structure. We provided an overview of recent findings concerning DSB formation, distribution and regulation, all of which will help us to determine whether meiotic DSB formation is evolutionarily conserved or varies between plants and other organisms.

## 1. Introduction

In flowering plants, reproductive cells develop in the ‘sporophytic generation’ and then differentiate into the gamete-forming ‘gametophytic generation’ [1,2]. During the reproductive process, the production of gametes requires that the genetic complement be reduced by one-half [3,4,5]. This specialized nuclear division, called meiosis, includes one round of DNA replication followed by two successive rounds of cell division, thereby ensuring the ploidy of the zygotic genome [6,7]. In the first meiotic division (meiosis I), homologous chromosomes are brought in close to pair and undergo synapsis, promoting the reciprocal exchange of parental chromosome fragments and thereby increasing the genetic diversity of the progeny [7,8,9]. Meiotic recombination is initiated by the formation of programmed DNA double-strand breaks (DSBs) catalyzed by the evolutionarily conserved type II topoisomerase–like enzyme SPO11 and several accessary proteins [9,10,11,12,13]. After resection, two SPO11 molecules remain covalently bound to each 5′ end of the nicked DNA, which is then processed by the MRX complex (Mre11-Rad50-Xrs2) with the cooperation of Sae2 to release the SPO11-bound nicked DNA oligonucleotide [14,15,16,17,18,19]. Following the action of the 5′ to 3′ exonuclease Exo1, the DNA ends are further degraded to produce 3′ single-stranded DNA tails [9,20,21,22]. The RecA family recombinases RAD51 and DMC1 bind these single-stranded DNA tails, generating DNA-protein filaments that search and invade the homologous duplex DNA [23,24,25,26,27,28,29,30,31,32,33], resulting in the formation of the recombination intermediate D-loop structure, followed by DNA repair and synthesis [34,35,36]. Consequently, the D-loop can be processed via different pathways to yield either crossovers or non-crossovers [37,38,39,40,41]. In the past decades, a growing body of knowledge related to meiotic DSB initiation has been obtained from various plant species. Although the core molecular processes that generate DSBs are largely conserved between plants and other species, the exact mechanisms involved vary among species. In this review, we introduced a generalized framework for the key players and regulatory pathways that produce DSBs and compare the conserved and non-conserved mechanisms of recombination initiation between plants and other species.

## 2. Conserved Spo11 and Non-Conserved Spo11 Accessary Proteins in Different Species

The programmed induction of DSBs along chromosomes is catalyzed by Spo11, which is a homolog of the A subunit of an archaeal topoisomerase (TopoVI), and this is a general mode for initiating meiotic recombination in fungi, invertebrates, mammals, and plants [4,42]. However, Spo11 does not act alone, as many other accessory proteins participate in the catalysis (Table 1). In *Saccharomyces cerevisiae* (*S. cerevisiae*), nine DSB accessory proteins have been characterized, namely Ski8, Rec114, Mei4, Mer2, Rec102, Rec104, Mre11, Rad50 and Xrs2 [4]. Ski8, a direct partner of Spo11, acts as a scaffold protein that recruits other DSB proteins to meiotic chromosomes. Rec114-Mei4-Mer2 constitutes a functional subgroup that promotes DSB formation at chromosome axes [43,44,45,46]. Rec102 and Rec104 form a subcomplex to bridge the Rec114-Mei4-Mer2 subcomplex and the Spo11–Ski8 subcomplex [44,47,48,49]. The MRX subcomplex, consisting of Mre11, Rad50 and Xrs2, is not only essential for break end resection, but is also involved in DSB formation via interaction with Mer2 [50,51,52].

In *Schizosaccharomyces pombe* (*S. pombe*), seven DSB accessory proteins have been identified in addition to the Spo11 ortholog Rec12 [4]. Some of these proteins have homologs in *S. cerevisiae*, such as Rec14 (Ski8), Rec24 (Mei4), Rec7 (Rec114) and Rec15 (Mer2), while the other three, Rec6, Rec10 and Mde2, have no clear homologs in *S. cerevisiae* or other organism [53,54,55,56,57,58]. The protein subcomplex is organized in both similar and dissimilar ways between *S. cerevisiae* and *S. pombe*. Rec12, Rec14 and Rec6 form the DSB catalytic core (commonly known as DSBC) in *S. pombe* [55]. Rec7, Rec24 and Rec15 assemble into the SFT (seven-fifteen-twenty four) subcomplex, and the interrelations among these partners are dependent on Rec10, which is the Red1 homolog in *S. cerevisiae* [55,56,59,60]. Although Rec10 is essential for DSB formation in *S. pombe*, Red1 is dispensable in *S. cerevisiae* [4]. Mde2 stabilizes the SFT complex via interaction with Rec15 at DSB sites. Interestingly, the Rad32-Rad50-Nbs1 complex, which corresponds to MRX in *S. cerevisiae*, is only required for DSB repair and not for DSB induction in *S. pombe* [61].

In mice, except for the conserved protein SPO11, another five proteins are indispensable for DSB formation, including REC114, MEI4, IHO1, MEI1 and TOPOVIBL [4,62,63]. The subcomplex comprising REC114, MEI4 and IHO1 is analogous to Rec114-Mei4-Mer2 in *S. cerevisiae*, and this subcomplex co-localizes with chromosome axes via the interaction between IHO1 and HORMAD1, which are key components of the cohesin complex [64,65,66]. MEI1 is a scaffold protein that assists with the localization of MEI4 at chromosome axes [67,68,69]. TOPOVIBL, encoding the TopoVIB subunit of TopoVI DNA topoisomerase, interacts with SPO11 to form a canonical TopoVI catalytic complex that induces DSBs [70].

The model plant *Arabidopsis thaliana* (*Arabidopsis*) has three Spo11 homologs, namely AtSpo11-1, AtSpo11-2 and AtSpo11-3. Two of them, AtSpo11-1 and AtSpo11-2, are essential for DSB formation [11,71,72,73,74], whereas AtSpo11-3 is not required for meiosis [75,76]. TopoVIB was recently identified to mediate the formation of the AtSpo11-1/AtSpo11-2 heterodimer in *Arabidopsis*, explaining the non-redundant nature between AtSpo11-1 and AtSpo11-2 [13,77]. In addition, AtPRD1, AtPRD2 and AtPRD3 have been demonstrated to be indispensable for DSB formation, whereas their functional mode in catalyzing DSB formation remain elusive [12,78,79]. By performing sophisticated in silico studies [4,8,78], the orthologs of AtPRD1, AtPRD2 and AtPRD3 have been suggested to be Mei1 in mice, Mei4 in yeast and Mer2 in yeast, respectively [12,80,81]. However, these proteins exhibit enormous sequence divergence with their orthologs in yeast or mice. Moreover, AtDFO encodes a plant-specific protein without any known conserved domain, and no homolog has been identified outside the plant kingdom [82].

In rice, six DSB-forming proteins have been identified so far, namely OsSPO11-1, OsSPO11-4, OsMTOPVIB, OsSDS, OsCRC1 and P31^comet^/OsBVF1. Although the participation of OsSPO11-1, OsSPO11-4 and OsMTOPVIB in DSB formation is conserved, as is their homologs in other species [83,84,85,86], the homologs of OsSDS and OsCRC1 in *Arabidopsis*, which are SDS and PCH2, respectively, are not required for DSB formation [87,88]. These findings indicate that the DSB-forming machinery has substantially diverged between monocot and dicot plants. Moreover, P31^comet^ was first identified to be involved in the spindle assembly checkpoint in human cells [89]. However, the rice P31^comet^, also named OsBVF1 in an independent study, was unambiguously demonstrated to be required for DSB formation [90,91]. Taken together, these results indicate that, except for Spo11 proteins, few of the other Spo11 accessory proteins are conserved at the sequence or functional level across the eukaryotic kingdoms.

## 3. Defining Meiotic DSB Hotspots in Different Species

Meiotic DSBs are not randomly distributed along eukaryotic chromosomes; rather, they are concentrated within discrete regions described as DSB hotspots [92]. To generate a high-resolution physical map of the meiotic DSB landscape, several methodologies have been established over the past three decades. The early method based on gel electrophoresis was used to measure DSBs at low efficiency in yeast and mouse [93,94,95,96,97]. Recently, mapping eukaryotic DSB landscapes has been momentously intensified with advances in intricate technologies, including SPO11-oligo mapping and single-stranded DNA sequencing (SSDS). SPO11-oligo mapping is achieved by immunoprecipitating tagged-Spo11 bound with oligonucleotides, which subsequently go through end-labeling, purification and sequencing [98]. This method has been effectively applied in several yeast species, mouse and *Arabidopsis* (Table 2) [92,99,100,101,102]. SSDS takes advantage of an antibody specifically recognizing either one of two DNA recombinases, RAD51 or DMC1, and utilizes chromatin immunoprecipitation to enrich single-stranded DNA that has undergone single-end invasion. With the high-throughput sequencing of enriched single-stranded DNA, the genome-wide distribution of DSB hotpots was successfully obtained in maize, mouse and human [103,104,105] (Table 2).

DSB hotspot designation is governed by a hierarchy of interrelated factors, including *cis*-regulatory elements, chromatin accessibility and high-order chromosome architecture (Table 2) [106]. Of particular prominence is the striking presence of DSB hotspots in repetitive DNA, such as transposon elements, although this aspect varies among species [10]. In maize, only ~25% of DSB hotspots have been discovered near genes, and the remaining hotspots are distributed in repetitive DNA—predominantly in Gypsy retrotransposons [105]. Although the *Arabidopsis* genome has fewer transposons compared with maize, a proportion of DSB hotspots were identified in Helitron/Pogo/Tc1/Mariner/MuDR DNA transposons [99]. Similarly, although most DSB hotspots tend to occur in genic regions, a significant overlap have been observed within the MULE-MuDR, TcMar-Mariner, hAT-Charlie and PiggyBac transposon families [107]. Moreover, DSB hotspots are evident in Ty retrotransposons in budding yeast. What is well established is that the meiotic recombination within or adjacent to repetitive elements is rare because this could lead to homologous recombination between non-allelic repeats, likely resulting in harmful chromosomal rearrangements and compromising genome stability [108,109]. Therefore, the paradox between the appearance of DSB hotspots and the suppression of recombination in these repetitive regions suggests that a mechanism exists to safeguard the repair of DSB in repetitive regions and prevent inadvertent crossing over.

## 4. Control of Meiotic DSB Formation by Protein Phosphorylation

### 4.1. Cyclin-Dependent Kinases (CDKs)

**Figure 1:**
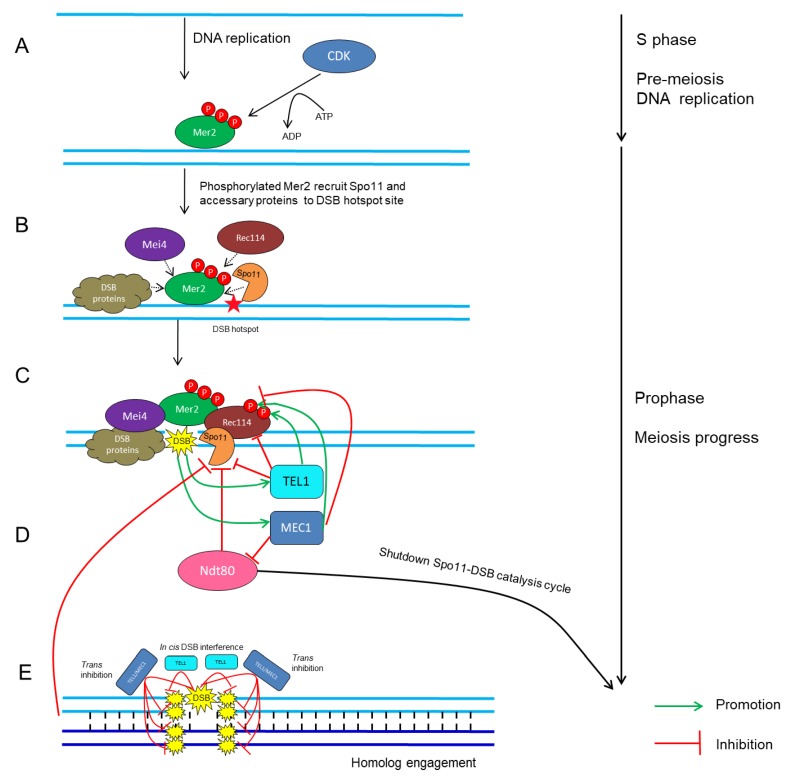
Schematic network of CDK- and ATM/ATR-mediated regulatory cycles of meiotic DSB timing and number in *S. cerevisiae*. (**A**) CDK phosphorylates Mer2 during pre-meiotic DNA replication [114,115,120]. (**B**) Phosphorylated Mer2 recruits Rec114, Mei4, Spo11 and other DSB proteins to DSB hotspot sites [45,120,121]. (**C**) DSB formation catalyzed by Spo11 and accessary proteins [4,66]. (**D**) Recurrent DSB formation activates TEL1/MEC1-dependent positive- and negative-feedback loops, which then restrains Spo11 activity and regulates the rate and number of DSB formation [136,140,147,156]. (**E**) *Cis* DSB interference mediated by TEL1 reduces the frequency of coincident DSB formation at the region adjacent to an already-formed DSB [143,149]. *Trans* inhibition mediated by TEL1 and MEC1 describes the ability of a DSB formation on one chromosome to suppress DSB formation on its homolog and sister chromatid at the same or adjacent regions [141].

CDKs in conjunction with their cyclin partners represent an ancient molecular switch that promotes and regulates cell-cycle progression [4,114,115]. The fundamental theme of how CDKs mediate meiotic recombination initiation was mostly drawn from studies in yeast. In *S. cerevisiae*, the activation of Cdc28 by its two B-type cyclin partners, Clb5 and Clb6, stimulates the phosphorylation on Mer2 during pre-meiotic DNA replication (Figure 1A) [115,116,117,118,119]; subsequently, phosphorylated Mer2 recruits other Spo11-accessary proteins to initiate DSB formation (Figure 1B) [45,120,121]. Similarly, in *S. pombe*, the association between Cdc2 and any of the three cyclins, Crs1, Cig1 and Cig2, is crucial for DSB formation [114].

In *Arabidopsis*, there are at least five types of cell-cycle CDKs (CDKA;1, CDKB;1, CDKB1;2, CDKB2;1 and CDKB2;2) and more than 50 cyclins [122,123,124,125,126,127,128,129,130,131], of which a few have been characterized as functioning during meiosis. CDKA;1 was originally identified as the key regulator for both mitosis and meiosis progression in *Arabidopsis* [129,131,132], and very recently, CDKA;1 was identified as a major regulator of meiotic recombination by mediating the number and placement of crossovers of homologous chromosomes [124,131]. However, CDKA;1 seems not to regulate meiotic prophase I although the exact influence on DSB formation was not determined in that study [124]. SDS, a cyclin protein, has been shown to control the formation of crossovers, but not DSBs, which differs from the necessity of its rice homologs in DSB formation [87,127,132,133]. TAM, an A-type cyclin (CYCA1;2), is indispensable for meiosis termination at the end of the first meiotic division [134,135]. Therefore, the core CDKs or related cyclins that directly regulate DSB formation remain to be explored in plants.

### 4.2. Tel1/ATM and Mec1/ATR

Once DSBs are made, there are several mechanisms to maintain the proper number of DSBs within a chromosome region [95,111,136,137,138,139,140,141]. This phenomenon, termed DSB homeostasis, prevents the severe effects caused by excess or insufficient Spo11 activity [136,137,140,142,143]. In *S. cerevisiae*, Tel1 and the related protein kinase Mec1 act as key factors that synergistically fine-tune the number of DSBs [141,142,144]. Lack of Tel1 leads to a considerable increase in the level of Spo11-oligonucleotide complexes, which are the by-product of meiotic DSB formation, indicating that Tel1 negatively regulates DSB formation (Figure 1C) [95]. In contrast, a defect in Mec1 activity causes a dramatic reduction in DSB number via inhibition of Ndt80, which triggers the exit from pachytene and shuts down Spo11-DSB catalysis of the cell cycle [145,146]. Mec1 ensures persistent Spo11 activity and indirectly promotes DSB formation, revealing that Mec1 positively regulates DSB formation (Figure 1C) [147,148]. In addition, recent studies in *S. cerevisiae* have revealed a *cis*-regulatory machinery, termed DSB interference, that reduces the frequency of coincident DSBs at the region adjacent to the preexisting DSB [136,142,143], and this process is dependent on TEL1 over short distances of ~70–100 kb (Figure 1D) [95,149]. On the contrary, the other machinery, known as *trans*-inhibition, defines the ability of a DSB to occur on a single chromosome, and this suppresses DSB designation on its homolog and sister chromatid at the same locus [141]. This mechanism relies on both Tel1 and Mec1, ensuring that an interhomolog interaction surrounding a DSB will not occur twice at the allelic or two nearby chromosomal positions so that DSBs are constrained to one per pair of homologs (Figure 1E) [141]. Interestingly, homolog engagement can also restrict the number of DSBs by inhibiting Spo11 activity (Figure 1E) [101,150]. Moreover, Tel1 and/or Mec1 can phosphorylate Rec114 directly, which limits its interaction with DSB hotspots and consequently reduces DSB formation genome wide (Figure 1C) [140].

In *Arabidopsis*, Tel1 and Mec1 homologs exist, termed ATM and ATR, respectively. However, ATM and ATR play synergetic roles in maintenance of genomic stability in meiotic cells by processing Spo11-dependent DSBs rather than influencing DSB formation [151,152,153,154,155]. Therefore, either ATM/ATR-mediated signaling pathway is evolutionarily divergent across different species or the effects of ATM/ATR on DSB formation is too subtle to be detected by conventional methods.

## 5. Control of Meiosis DSB Formation in the Context of ‘Tethered Loop-Axis Complex’

**Figure 2:**
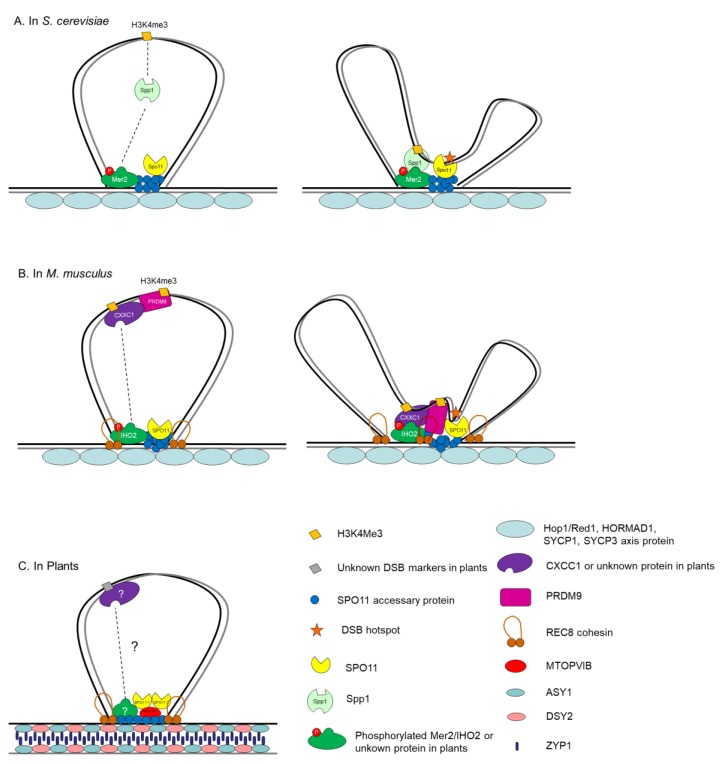
Different ‘tethered loop–axis complex’ models in *S. cerevisiae*, *M. musculus* and plants. (**A**) In *S. cerevisiae*, Spp1 recognizes and binds to DSB hotspots adjacent to H3K4me2/3 on loops via interaction with axis-bound Mer2 [55,161,162]. (**B**) In *M. musculus*, CXXC1 interacts with PRDM9 and IHO2, which designates DSB hotspots in loops and is located on chromosome axes, respectively; this interaction tethers the chromatin loop to the axis for DSB formation [158,163,164,165,166]. (**C**) In plants, the major players involved in bridging chromatin loops with axis remain uncharacterized [88,91,167,168,169].

Spatiotemporal control of DSB formation takes place within a specialized chromosomal structure featuring replicated sister chromosomes organized into linear looped arrays that emanate from a central proteinaceous axis [4,66,106,157,158]. However, DSBs are known to occur primarily on DNA sequences in the loops, whereas most of the Spo11 accessory proteins are located on the axis [43,44,45,66,159]. To reconcile this spatial inconsistency, different species have adapted a similar ‘tethered-loop/axis complex’ system in which DSB loci within chromatin loops can be tethered to the chromosome axis by DSB-promoting factors [66,157,160], although key players in this system are evolutionarily divergent across various species [158]. In *S. cerevisiae*, for example, Spp1, a PHD finger domain protein and Set1 COMPASS complex member, binds to H3K4me2/3 adjacent to DSB-prone sites on loops and interacts transiently with axis-bound Mer2, establishing a spatial linker between DSB loci and the DSB-forming machinery, thus promoting DSB formation on chromosome axes (Figure 2A) [158,161,162]. In mouse, PRDM9, the meiosis-specific histone methyltransferase, defines potential DSB sites through catalyzing trimethylation of H3K4 [163], and via its KRAB domain interacts with CXXC1, the ortholog of *S. cerevisiae* Spp1 [158,164], which correspondingly interacts with IHO1, a DSB-promoting protein located on chromosome axes (Figure 2B) [165,166]. 

In plants, although the formal ‘tethered-loop/axis complex’ system remains to be established, there are many indications that this may also be the case. Recently, using super-resolution microscopy, Spo11-1 and DSBs sites were found to be associated with chromosome axes in maize [167]. Additionally, several structural components of the axial elements have been demonstrated to be required for maintaining the normal number of DSB formation, such as ASY3 in *Arabidopsis* [168], CRC1 and P31^comet^ in rice [88,91] and DSY2 in maize [169]. However, the direct factors bridging chromosome axis components and the DSB-forming machinery await characterization (Figure 2C). 

## 6. Concluding Remarks

In recent years, much progress has been made toward understanding meiotic DSB initiation and distribution in plants. These advances allowed researchers to translate acquired knowledge from model species to various crops, which facilitated breeding programs [170]. However, although commonalities exist, different strategies and mechanisms for the spatiotemporal initiation and regulation of DSB formation have evolved for diverse plant species, primarily owing to differences in genome size and chromatin organization. Recent innovations in CRISPR/Cas9-mediated gene editing, as well as high-throughput sequencing have greatly accelerated functional genomics studies in many important crop species with more complex genomes, including polyploidy. Therefore, additional studies of meiotic genes that regulate the initiation of recombination in these plant species would deepen our understanding on how the designation and distinction of DSBs diverged between species from the evolutionary aspect.

## Figures and Tables

**Table 1:** Characterized proteins involved in meiotic double-strand breaks (DSB) formation in different organisms.

*Arabidopsis Thaliana*	*Oryza Sativa*	*Saccharomyces Cerevisiae*	*Schizosaccharomyces Pombe*	*Mus Musculus*
DFO				
PRD1				MEI1
PRD2		Mei4	Rec24	MEI4
PRD3	PAIR1	Mer2/Rec107	Rec15	IHO1
Spo11-1,2	Spo11-1,4	Spo11	Rec12	SPO11
TopoVI B	TopoVI B			TopoVI B
	P31^comet^/BVF1			P31^comet^
SDS	SDS			
-	CRC1			
		Rec102		
		Rec104		
			Rec6	
		Rec114	Rec7	REC114
			Rec10	
		Ski8/Rec103	Rec14	WDR61
			Mde2	
MRE11		Mre11	Rad32	
RAD50		Rad50	Rad50	RAD50
NBS1		Xrs2	Nbs1	NBS1

**Table 2:** Meiotic DSB hotspots identified in different species by SPO11-oligo mapping or single-stranded DNA sequencing (SSDS).

Species	Genome Size	Chromosome No.	Number of DSBs	DSB Hotspot No.	Most Common DSB Location	Average Width (kb)	Predominantly DSB Formation Among Transposon	Method	Hotspot Detection	References
*S. cerevisiae* (SK1)	12.1 Mb	16	~175	3604–4099	Gene promoters	0.248–0.264	Ty retrotransposons	SPO11-oligos	Enrichment threshold	[92,101,110,111]
*S. cerevisiae* (YPS128)	12.1 Mb	16	~175	4177	Gene promoters	0.265	n/a	SPO11-oligos	Enrichment threshold	[112]
*S. cerevisiae* (UWOPS03-461.4)	12.1 Mb	16	~175	3881	Gene promoters	0.256	n/a	SPO11-oligos	Enrichment threshold	[112]
*S. pombe*	13.8 Mb	3	~60	603	All chromosome regions	1.4	n/a	Rec12-oligos	Enrichment threshold	[113]
*M. musculus* (9R×13R)	2.8 Gb	20	~250	9874– 15,677	Intergenic	~2.000–3.400	LTR retrotransposons SINE	SSDS	Peak calling	[103,104]
*M. musculus* (9R)	2.8 Gb	20	~250	14,869	Intergenic	~2.000	n/a	SSDS	Peak calling	[104]
*M. musculus* (13R)	2.8 Gb	20	~250	15,481	Intergenic	~2.000	n/a	SSDS	Peak calling	[104]
*M. musculus* (B6)	2.8 Gb	20	~250	18,313	Intergenic	~2.000	n/a	SSDS	Peak calling	[104]
*M. musculus* (B6)	2.8 Gb	20	~250	13,960	Intergenic	~0.281	n/a	SPO11-oligos	Enrichment threshold	[100]
*Arabidopsis thaliana*	135 Mb	5	~250–300	5914	Gene promoters and terminators	0.823	Helitron /Pogo/Tc1/Mariner DNA transposons	SPO11-1-oligos	Peak calling	[99]
*Zea mays*	2.4 Gb	10	~500	3126	All chromosome regions	1.2	Gypsy retrotransposons	SSDS	Peak calling	[105]
